# Correction: BMDD: A probabilistic framework for accurate imputation of zero-inflated microbiome sequencing data

**DOI:** 10.1371/journal.pcbi.1013974

**Published:** 2026-02-17

**Authors:** Huijuan Zhou, Jun Chen, Xianyang Zhang

The Funding statement for this article is incorrect. The correct Funding statement is as follows: This work was supported by the National Institutes of Health (R01GM144351 to JC & XZ); the National Science Foundation (DMS-1830392, DMS-2113359, DMS-1811747 to XZ; DMS-2113360 to JC); and the National Natural Science Foundation of China (12201384 to HZ). The funders had no role in study design, data collection and analysis, decision to publish, or preparation of the manuscript. The primary work was conducted in the United States with NIH/NSF support. After returning to China, HZ completed additional work using non-U.S. funds; no NIH funds supported work performed outside the United States
