# Supplementary material for: BMDD: A probabilistic framework for accurate imputation of zero-inflated microbiome sequencing data
Source: PLoS Comput Biol. 2025 Oct 24;21(10):e1013124. doi: 10.1371/journal.pcbi.1013124 (PMC12582502; doi:10.1371/journal.pcbi.1013124)
Supplement: S1 File — Fig A1. Posterior predictive check for the four example taxa from the American Gut Project. The blue histogram shows the observed data, and the red histogram represents data generated by sampling from multinomial distributions parameterized with posterior samples of the compositions from BMDD. “0s” indicates the proportion of zeros, and “SD” denotes the standard deviation.Fig A2. Performance of ANCOM-BC with BMDD on the four real datasets. (Bottom) Number of discoveries vs. target FDR level for the real datasets; (Top) Empirical FDR vs. target FDR level for the shuffled real datasets. The dashed gray line represents the diagonal. The results were averaged over 100 simulation runs.Fig A3. A simplified illustration of the phylogenetic tree. ◯ denotes internal node while △ represents leaf node.Table A1. Simulation results for true composition estimation under setting S1 (BMDD model). The values outside the parentheses are estimates of the corresponding evaluation metrics achieved by each method (averaged over 10 simulation runs), and the values in parentheses are standard errors. Bold red and red values in each row indicate the two best methods for each evaluation metric.Table A2. Characteristics of the real microbiome datasets. The second to fifth columns respectively list the number of taxa, sample size, numbers of the controls and cases of each filtered dataset (prevalence ≥ 20%, library size ≥ 1000). (PDF) [file pcbi.1013124.s001.pdf]

Supplementary Materials (S1 File)

A1 Posterior predictive check of BMDD

Figure A1 presents the results of posterior predictive check for the four example taxa from the American Gut Project.

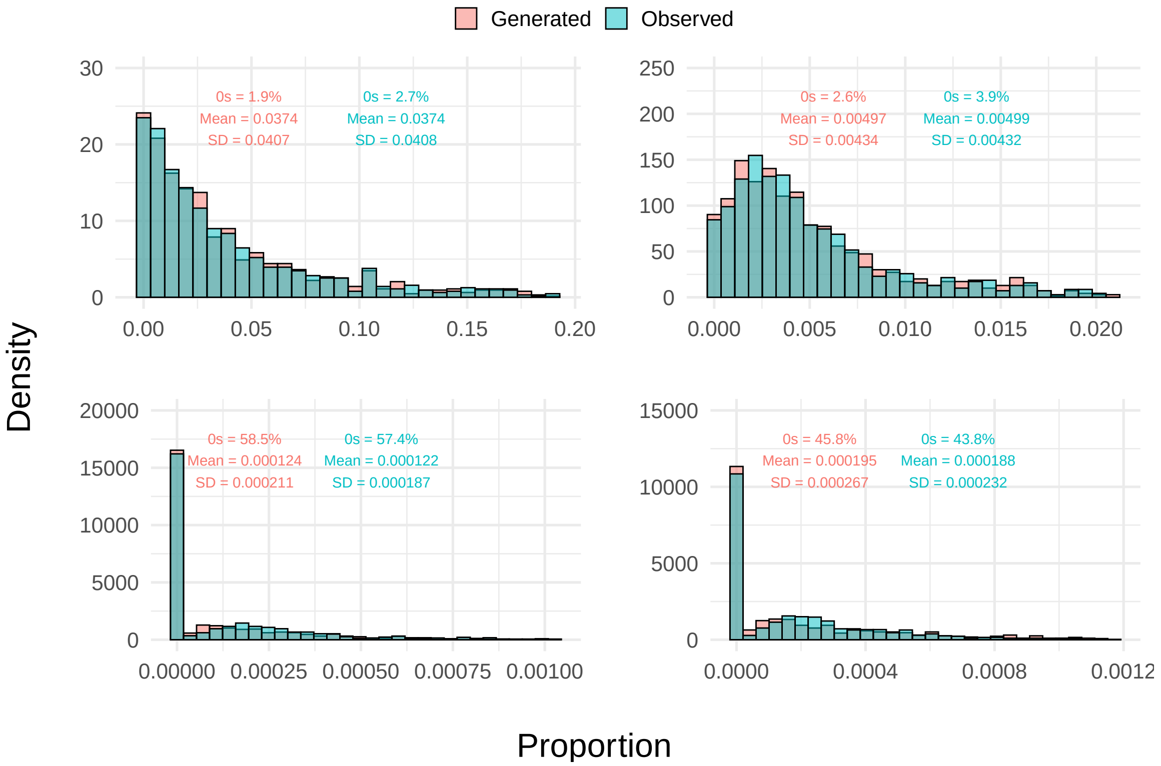

Figure A1: Posterior predictive check for the four example taxa from the American Gut Project. The blue histogram shows the observed data, and the red histogram shows data generated by sampling from multinomial distributions parameterized with posterior samples of the compositions from BMDD. "0s" indicates the proportion of zeros, and "SD" denotes the standard deviation.

A2 Detailed setups and additional results for true composition estimation

A2.1 Detailed simulation setups

S1: Data generated from the BMDD model. We examined the setting where data were generated from our theoretical model, BMDD,

$$Y_{i,j}|\theta_{ij} \sim \text{Gamma}(\theta_{ij}, 1), \tag{A1}$$

where θ_{ij} is a random variable with point masses at $\alpha_{j,0}$ and $\alpha_{j,1}$, as defined in formula (1) of the main text. The parameters π_j, $\alpha_{j,0}$ and $\alpha_{j,1}$ were estimated by fitting BMDD to the COMBO dataset from a cross-sectional study of the diet effect on stool microbiome composition (Wu et al. 2011). The full source code and detailed parameter values are available at https://github.com/zhouhj1994/BMDD-manuscript-sourcecode.

S2–S5: Data generated by alternative models with correlations. Microbial taxa coexist in functionally related ways, and therefore, their abundances may display covariance. To mimic the dependency of abundances across taxa, we introduced several correlation structures into the data. In addition to the gamma model (A1) to generate true absolute abundance, we also considered three other commonly used probabilistic models :

$$\log(Y_{i,j})|\theta_{ij} \sim N(\theta_{ij}, 2), \tag{A2}$$
$$Y_{i,j}|\theta_{ij} \sim \text{Poisson}(\theta_{ij}), \tag{A3}$$
$$Y_{i,j}|\theta_{ij} \sim \text{NB}(0.5, 0.5/(0.5 + \theta_{ij})), \tag{A4}$$

for log-normal, Poisson, and negative binomial, respectively, where $\text{NB}(a, b)$ represents the distribution of the number of failures in a sequence of Bernoulli trials with the target number of successes a and probability of success $b \in (0, 1)$. Settings S2–S5 correspond to the taxa distributions given in equations (A1–A4).

- Correlation structure. We set $m = 100$ and designated 30 taxa to have bimodal composition distributions with equal probability of being from each mode and 70 taxa to have a single mode, labeling them as 1–100. We included several types of correlations: (i) Taxa 1–5 are simultaneously generated from either the larger or smaller mode in each sample, while taxa 6–10 are generated from the opposite mode; (ii) When taxa 11–12 are drawn from the larger mode, taxa 13–16 are also drawn from the larger mode, whereas taxa 17–20 are drawn from the smaller mode. Additionally, the single-modal taxa 31–35 are set to be abundant by resetting

  $$Y_{i,j} \sim \text{Unif}(\text{Quantile}(\boldsymbol{Y}_{(j)}, 0.75), \max \boldsymbol{Y}_{(j)})$$

  for $j = 31, \ldots, 35$ and $i \in \{i = 1, \ldots, n : \delta_{i,11} = \delta_{i,12} = 1\}$, where $\boldsymbol{Y}_{(j)} = (Y_{1,j}, \ldots, Y_{n,j})$, $\text{Quantile}(\boldsymbol{y}, q)$ represents the $q \times 100\%$ quantile, and $\max \boldsymbol{y}$ represents the maximum value of sample $\boldsymbol{y}$. Similarly, taxa 36–40 are set to be rare by resetting

  $$Y_{i,j} \sim \text{Unif}(\min \boldsymbol{Y}_{(j)}, \text{Quantile}(\boldsymbol{Y}_{(j)}, 0.25)),$$

  where $\min \boldsymbol{y}$ represents the minimum value of sample $\boldsymbol{y}$; (iii) If taxon 41 has an absolute abundance exceeding its third quartile across samples, that is, $Y_{i,41} > \text{Quantile}(\boldsymbol{Y}_{(41)}, 0.75)$, then taxa 42–45 are set to be abundant, whereas taxa 46–50 are set to be rare; (iv) Taxa 51–60 increase across samples, that is $Y_{1,j} \leq Y_{2,j} \leq \cdots \leq Y_{n,j}, j = 51, \ldots 60$, whereas taxa 61–70 decrease across samples.

S6–S9: Data generated by non-parametric models. Given a real count matrix, we randomly selected n samples and m taxa from the matrix and regarded it as the true absolute abundance. We generated the observed count matrix by randomly masking (replacing with zeros) a part of non-zero values in the original count matrix to further increase zero

inflation to make the estimation more challenging. The masking probability function was estimated in a similar way as in Arisdakessian et al. (2019). Specifically, for each taxon, we extracted the proportion of zeros vs. the mean of those positive values; then, we fitted a logistic function to these data points. We masked one-fifth of the non-zero values of each taxon by random sampling with the probability weights given by the logistic function previously fitted. We used four datasets as the baseline from the studies of Clostridium difficile infection (CDI) (Schubert et al. 2014), inflammatory bowel disease (IBD) (Morgan et al. 2012), rheumatoid arthritis (RA) (Scher et al. 2013), and smoking effect on the human upper respiratory tract (SMOKE) (Charlson et al. 2010). The settings with the four datasets are referred to as S6–S9 respectively.

For the parametric models, we let the total counts N_i be independently generated from NB(0.5, 0.5 / (0.5 + 1000)), i.e., the negative binomial distribution with dispersion parameter 0.5 and mean 1000. In addition, we set the composition $X_{i,j}$ to be 0 if its original value is smaller than 10^{-6} to generate physically absent taxa and then normalized the data to ensure that the composition sums up to 1. Based on the true compositions and the total counts, multinomial models are used to generate the observed counts.

A2.2 Evaluation metrics

The specific formulations of the evaluation metrics introduced in Section 2.2 in the main text are given below. Let $\widehat{\boldsymbol{X}}$ denote the estimate of the true composition matrix. Let $\boldsymbol{X}_i = (X_{i,1}, \ldots, X_{i,m})$, $i = 1, \ldots, n$, denote the composition of ith sample, and $\boldsymbol{X}_{(j)} = (X_{1,j}, \ldots, X_{n,j})$, $j = 1, \ldots, m$, denote the proportions of jth taxon across all samples.

General similarity measures between two composition matrices

1. Mean squared error (SE):

$$M_{se}(\widehat{\boldsymbol{X}}, \boldsymbol{X}) = \frac{1}{mn} \sum_{i=1}^{n} \sum_{j=1}^{m} (X_{i,j} - \widehat{X}_{i,j})^2.$$

2. Sample-wise distance (SD):

$$M_{sd}(\widehat{\boldsymbol{X}}, \boldsymbol{X}) = \frac{1}{n} \sum_{i=1}^{n} \|\boldsymbol{X}_i - \widehat{\boldsymbol{X}}_i\|_2.$$

3. Taxon-wise distance (TD):

$$M_{td}(\widehat{\boldsymbol{X}}, \boldsymbol{X}) = \frac{1}{m} \sum_{j=1}^{m} \|\boldsymbol{X}_{(j)} - \widehat{\boldsymbol{X}}_{(j)}\|_2.$$

Preservation of sample-wise properties

1. Shannon's index (SH): Let $H_{sh}(\boldsymbol{X}_i) = -\sum_{j=1}^{m} X_{i,j} \log X_{i,j}$,

$$M_{sh}(\widehat{\boldsymbol{X}}, \boldsymbol{X}) = \frac{1}{n} \sum_{i=1}^{n} \left(H_{sh}(\widehat{\boldsymbol{X}}_i) - H_{sh}(\boldsymbol{X}_i) \right)^2.$$

2. Simpson's index (SP): Let $H_{sp}(\boldsymbol{X}_i) = \sum_{j=1}^{m} X_{i,j}^2$,

$$M_{sp}(\widehat{\boldsymbol{X}}, \boldsymbol{X}) = \frac{1}{n} \sum_{i=1}^{n} \left(H_{sp}(\widehat{\boldsymbol{X}}_i) - H_{sp}(\boldsymbol{X}_i) \right)^2.$$

3. Bray–Curtis dissimilarity (BC): Let $\widetilde{\boldsymbol{X}}_i = \boldsymbol{X}_i \wedge \widehat{\boldsymbol{X}}_i$, where $\wedge$ means taking lesser values between the two vectors elementwisely, $\mathrm{BC}(\boldsymbol{X}_i, \widehat{\boldsymbol{X}}_i) = 1 - \sum_{j=1}^{m} \widetilde{X}_{i,j}$,

$$M_{bc}(\widehat{\boldsymbol{X}}, \boldsymbol{X}) = \frac{1}{n} \sum_{i=1}^{n} \mathrm{BC}(\widehat{\boldsymbol{X}}_i, \boldsymbol{X}_i).$$

4. Kullback–Leibler divergence (KL): Let $\mathrm{KL}(\boldsymbol{X}_i, \widehat{\boldsymbol{X}}_i) = \sum_{j=1}^{m} X_{i,j} \log(X_{i,j}/\widehat{X}_{i,j})$,

$$M_{kl}(\widehat{\mathbf{X}}, \mathbf{X}) = \frac{1}{n} \sum_{i=1}^{n} \mathrm{KL}(\boldsymbol{X}_i, \widehat{\boldsymbol{X}}_i).$$

5. Jensen–Shannon divergence (JS): Let $\widetilde{\boldsymbol{X}} = (\boldsymbol{X} + \widehat{\boldsymbol{X}})/2$,

$$M_{js}(\widehat{\boldsymbol{X}}, \boldsymbol{X}) = \frac{1}{n} \sum_{i=1}^{n} \frac{1}{2} \left(\mathrm{KL}(\boldsymbol{X}_i, \widetilde{\boldsymbol{X}}_i) + \mathrm{KL}(\widehat{\boldsymbol{X}}_i, \widetilde{\boldsymbol{X}}_i) \right).$$

6. Hellinger distance (HD): Let $\mathrm{HD}(\boldsymbol{X}_i, \widehat{\boldsymbol{X}}_i) = \frac{1}{\sqrt{2}} \|\boldsymbol{X}_i^{1/2} - \widehat{\boldsymbol{X}}_i^{1/2}\|_2$, where $\boldsymbol{X}_i^{1/2}$ means taking the square root on each element,

$$M_{hd}(\widehat{\boldsymbol{X}}, \boldsymbol{X}) = \frac{1}{n} \sum_{i=1}^{n} \mathrm{HD}(\boldsymbol{X}_i, \widehat{\boldsymbol{X}}_i).$$

Preservation of taxon-wise properties

1. Gini coefficient (GI): Let $\mathrm{Gini}(\boldsymbol{y}) = 1 - \frac{2}{n-1} \left(n - \frac{\sum_{i=1}^{n} i y_i}{\sum_{i=1}^{n} y_i} \right)$ for a random sample $\boldsymbol{y}$ with values $y_1 \leq y_2 \leq \cdots \leq y_n$,

$$M_{gi}(\widehat{\boldsymbol{X}}, \boldsymbol{X}) = \frac{1}{m} \sum_{j=1}^{m} (\mathrm{Gini}(\boldsymbol{X}_{(j)}) - \mathrm{Gini}(\widehat{\boldsymbol{X}}_{(j)}))^2.$$

2. Pair of mean and standard deviation (MS): Let (a_j, b_j) and $(\hat{a}_j, \hat{b}_j)$ be the sample mean and standard deviation of $\boldsymbol{X}_{(j)}$ and $\widehat{\boldsymbol{X}}_{(j)}$ respectively,

$$M_{ms}(\widehat{\boldsymbol{X}}, \boldsymbol{X}) = \frac{1}{m} \sum_{j=1}^{m} (a_j - \hat{a}_j)^2 + (b_j - \hat{b}_j)^2.$$

3. Coefficient of variation (CV):

$$M_{cv}(\widehat{\boldsymbol{X}}, \boldsymbol{X}) = \frac{1}{m} \sum_{j=1}^{m} (b_j/a_j - \hat{b}_j/\hat{a}_j)^2.$$

4. Kolmogorov–Smirnov distance (KS): Let $\mathrm{KS}(\boldsymbol{X}_{(j)}, \widehat{\boldsymbol{X}}_{(j)}) = \sup_x |F_1(x) - F_2(x)|$, where F_1, F_2 denotes the empirical distribution functions of the first and the second sample respectively,

$$M_{ks}(\widehat{\boldsymbol{X}}, \boldsymbol{X}) = \frac{1}{m} \sum_{j=1}^{m} \mathrm{KS}(\boldsymbol{X}_{(j)}, \widehat{\boldsymbol{X}}_{(j)}).$$

5. Wasserstein distance (WS): Let $\mathrm{WS}(\boldsymbol{X}_{(j)}, \widehat{\boldsymbol{X}}_{(j)}) = \frac{1}{n} \sum_{i=1}^{n} |X_{(j)}^{(i)} - \widehat{X}_{(j)}^{(i)}|$, where $X_{(j)}^{(i)}$ represents the ith order statistic of sample $\boldsymbol{X}_{(j)}$,

$$M_{ws}(\widehat{\boldsymbol{X}}, \boldsymbol{X}) = \frac{1}{m} \sum_{j=1}^{m} \mathrm{WS}(\boldsymbol{X}_{(j)}, \widehat{\boldsymbol{X}}_{(j)}).$$

6. Pairwise taxon-to-taxon correlation (TT):

$$M_{tt}(\widehat{\boldsymbol{X}}, \boldsymbol{X}) = \frac{2}{m(m-1)} \sum_{j<k} (\mathrm{Corr}(\boldsymbol{X}_{(j)}, \boldsymbol{X}_{(k)}) - \mathrm{Corr}(\widehat{\boldsymbol{X}}_{(j)}, \widehat{\boldsymbol{X}}_{(k)}))^2.$$

A2.3 Additional results

Table A1 provides detailed values for each evaluation metric across different methods under simulation setting S1.

Table A1: Simulation results for true composition estimation under setting S1 (BMDD model). The values outside the parentheses are estimates of the corresponding evaluation metrics achieved by each method (averaged over 10 simulation runs), and the values in parentheses are standard errors. Bold red and red values in each row indicate the two best methods for each evaluation metric.

	BMDD	SAVER	naive-1	mbDenoise	mbImpute	scImpute	ALRA	DMM	naive-2	naive-3	naive-4
Mean squared error	**5.25e-05**(3.26e-06)	6.36e-05(4.00e-06)	3.24e-04(4.93e-05)	2.52e-04(1.05e-05)	1.79e-04(1.60e-05)	5.83e-04(8.58e-05)	4.94e-04(3.17e-05)	1.85e-04(8.59e-06)	1.12e-04(1.06e-05)	9.09e-05(7.96e-06)	3.00e-04(4.51e-05)
Sample-wise distance	**0.0569**(0.0014)	0.0600(0.0014)	0.0962(0.0054)	0.1541(0.0030)	0.1098(0.0038)	0.1936(0.0125)	0.2160(0.0078)	0.1329(0.0032)	0.0810(0.0037)	0.0702(0.0027)	0.0934(0.0052)
Taxon-wise distance	**0.0502**(0.0012)	0.0537(0.0012)	0.0972(0.0042)	0.1045(0.0026)	0.0836(0.0023)	0.1156(0.0043)	0.1314(0.0022)	0.0994(0.0030)	0.0636(0.0013)	0.0599(0.0013)	0.0941(0.0041)
Shannon's index	0.0432(0.0029)	**0.0344**(0.0036)	0.4549(0.0590)	0.4480(0.0244)	0.4387(0.0316)	0.3299(0.0584)	1.2053(0.1219)	0.3678(0.0287)	0.4426(0.0411)	0.2988(0.0305)	0.3450(0.0476)
Simpson's index	**1.10e-04**(1.75e-05)	2.01e-04(7.04e-05)	0.0168(0.0045)	0.0010(9.49e-05)	0.0018(2.91e-04)	0.0159(0.0047)	0.0034(3.82e-04)	5.49e-04(3.78e-05)	9.54e-04(1.75e-04)	6.24e-04(1.19e-04)	0.0140(0.0037)
Bray-Curtis dissimilarity	**0.1551**(0.0035)	0.1614(0.0033)	0.2009(0.0067)	0.4197(0.0096)	0.3102(0.0067)	0.3429(0.0124)	0.5499(0.0169)	0.3848(0.0116)	0.2231(0.0062)	0.1973(0.0054)	0.1962(0.0065)
Kullback–Leibler divergence	**0.1668**(0.0066)	0.1819(0.0065)	2.0603(0.1237)	0.6101(0.0197)	0.4156(0.0143)	1.3754(0.0705)	1.6758(0.1280)	0.5747(0.0228)	0.2758(0.0135)	0.2424(0.0119)	0.4740(0.0317)
Jensen–Shannon divergence	**0.0398**(0.0016)	0.0421(0.0014)	0.0748(0.0041)	0.1615(0.0047)	0.1129(0.0032)	0.1444(0.0067)	0.2387(0.0114)	0.1412(0.0055)	0.0724(0.0032)	0.0616(0.0028)	0.0663(0.0037)
Hellinger distance	**0.1784**(0.0036)	0.1833(0.0031)	0.2353(0.0064)	0.4366(0.0064)	0.3646(0.0051)	0.4013(0.0107)	0.5288(0.0151)	0.4021(0.0081)	0.2473(0.0059)	0.2191(0.0053)	0.2112(0.0058)
Gini coefficient	0.0091(9.49e-04)	0.0092(9.72e-04)	0.0102(9.39e-04)	0.2686(0.0065)	0.1438(0.0077)	**0.0031**(1.51e-04)	0.2122(0.0410)	0.2175(0.0108)	0.0497(0.0036)	0.0263(0.0024)	0.0055(5.87e-04)
(Mean, Standard deviation)	**5.61e-06**(5.47e-07)	1.05e-05(2.53e-06)	1.54e-04(3.49e-05)	1.80e-04(6.85e-06)	7.98e-05(8.38e-06)	2.01e-04(4.99e-05)	3.73e-04(3.56e-05)	7.55e-05(6.84e-06)	3.71e-05(5.14e-06)	1.70e-05(2.65e-06)	1.38e-04(3.12e-05)
Coefficient of variation	**0.0912**(0.0090)	0.1030(0.0095)	0.7197(0.0721)	1.9305(0.0525)	1.0623(0.0672)	0.1370(0.0149)	1.5584(0.2229)	1.6840(0.0717)	0.6372(0.0488)	0.4044(0.0403)	0.5757(0.0551)
Kolmogorov–Smirnov distance	0.2697(0.0054)	0.2762(0.0057)	0.4383(0.0075)	0.5678(0.0038)	0.4913(0.0102)	**0.1603**(0.0022)	0.5928(0.0355)	0.5632(0.0102)	0.3777(0.0084)	0.3198(0.0082)	0.3487(0.0065)
Wasserstein distance	**0.1308**(0.0044)	0.1372(0.0034)	0.1889(0.0089)	0.5593(0.0146)	0.4047(0.0104)	0.2342(0.0120)	0.7420(0.0406)	0.4463(0.0163)	0.2889(0.0110)	0.2141(0.0096)	0.1830(0.0086)
Pairwise taxon-to-taxon correlations	**0.0060**(3.80e-04)	0.0068(5.18e-04)	0.0116(6.17e-04)	0.3865(0.0043)	0.0133(7.18e-04)	0.0166(8.50e-04)	0.2288(0.0471)	0.1462(0.0111)	0.0216(0.0042)	0.0183(0.0038)	0.0114(6.03e-04)

Table A2: Characteristics of the real microbiome datasets. The second to fifth columns respectively list the number of taxa, sample size, numbers of the controls and cases of each filtered dataset (prevalence $\geq 20\%$, library size ≥ 1000).

	m	n	n (controls)	n (cases)	Reference
IBD-1	269	76	21	55	Papa et al. (2012)
IBD-2	245	86	36	50	Willing et al. (2010)
IBD-3	757	161	16	145	Gevers et al. (2014)
IBD-4	123	123	14	109	Morgan et al. (2012)

A3 Detailed setups and additional results for differential abundance analysis

A3.1 Detailed simulation setups

As in Zhou et al. (2022), we used the COMBO data as the reference dataset to estimate the parameters in the four data-generation models:

$$
\begin{aligned}
Y_{i,j} &\sim \text{Gamma}(a_j \exp(b_j u_i), 1),\\
\log(Y_{i,j}) &\sim N(a_j + b_j u_i, \sigma_j^2),\\
W_{i,j} &\sim \text{Poisson}(\exp(a_j N_i + b_j u_i)),\\
W_{i,j} &\sim \text{NB}(d_j, d_j/\{d_j + \exp(a_j N_i + b_j u_i)\}),
\end{aligned}
$$

where a_j, σ_j^2, and d_j were estimated based on the COMBO data, u_i, which was set to be a binary variable, is the variable of interest, N_i is the sequencing depth generated from the negative binomial distribution with parameters estimated from the COMBO data, and b_j was set to be either zero for non-differential taxon or non-zero for differential taxon. For gamma and log-normal distributions, we let $X_{i,j} = \frac{Y_{i,j}}{\sum_{k=1}^{m} Y_{i,k}}$ and generated the observed abundance from $W_{i,1}, \ldots, W_{i,m} \sim \text{Multinomial}(X_{i,1}, \ldots, X_{i,m})$ with $N_i = \sum_{j=1}^{m} W_{i,j}$.

A3.2 Characteristics of the real datasets

Table A2 summarizes the characteristics of the four real microbiome datasets.

A3.3 Detailed studies of the results for IBD-2, IBD-3 and IBD-4

We provide detailed analyses of the results for IBD-2, IBD-3, and IBD-4 (Figures 4b and 4c in the main text) below.

For IBD-2, all three methods controlled the FDR, with LinDA-SAVER making the most discoveries, followed by LinDA-BMDD and LinDA. At 10% FDR, LinDA-BMDD, LinDA-SAVER, and LinDA identified 3, 15, and 4 differential taxa, respectively. Two differential taxa identified by LinDA-BMDD were also identified by the other two methods, while the remaining one was also identified by LinDA-SAVER. Ten out of the fifteen differential taxa identified by LinDA-SAVER were absent from the results of both LinDA-BMDD and LinDA. For IBD-3, all three methods showed some FDR inflation, with LinDA-BMDD displaying the mildest FDR inflation and the fewest discoveries. At 10% FDR, LinDA-BMDD,

LinDA-SAVER, and LinDA identified 28, 83, and 48 differential taxa, respectively, with 25 differential taxa common to the three methods and 1, 40, and 3 unique to LinDA-BMDD, LinDA-SAVER, and LinDA, respectively. The one unique taxon to LinDA-BMDD, belonging to Blautia of Lachnospiraceae family, has been shown to be associated with IBD (Mah et al. 2023). For IBD-4, LinDA-BMDD made a similar number of discoveries to LinDA and fewer discoveries than LinDA-SAVER, and displayed the best FDR control compared to the other two methods. At 10% FDR, LinDA-BMDD, LinDA-SAVER, and LinDA identified 37, 55, and 42 differential taxa, respectively. Thirty-three differential taxa identified by LinDA-BMDD were also identified by either or both of the other methods. LinDA-BMDD, LinDA-SAVER, and LinDA had 4, 11, and 2 method-specific taxa, respectively. Two unique taxa identified by LinDA-BMDD belonged to Blautia of Lachnospiraceae family, one belonged to Clostridium XlVa of Lachnospiraceae family, and the remaining one belonged to Anaerofilum of Ruminococcaceae family. This result is consistent with our previous findings. The literature has also shown disruption in the taxa of the Lachnospiraceae and Ruminococcaceae families in IBD patients compared to controls (Schirmer et al. 2018, Yilmaz et al. 2019). Taken together, BMDD significantly increases the robustness of LinDA by providing a more effective false positive control. The results by LinDA-BMDD are expected to be more reproducible.

A3.4 Additional results

Figure A2 compares the performance of the original ANCOM-BC to ANCOMBC-BMDD and ANCOMBC-SAVAER on the four real datasets.

A4 Mixture regression modeling

A4.1 Incorporating sample covariates

In practice, we often observe a set of sample covariates (say $\boldsymbol{Z}_i \in R^k$) together with the taxa counts. Here, we introduce a mixture regression framework to incorporate these sample covariates. Specifically, we consider the following model:

$$
\begin{aligned}
&W_{i,1}, \ldots, W_{i,m} | \boldsymbol{X}_i \sim \text{Multinomial}(X_{i,1}, \ldots, X_{i,m}), \\
&X_{i,1}, \ldots, X_{i,m} | \boldsymbol{\theta}_i \sim \text{Dirichlet}(\theta_{i1}, \ldots, \theta_{im}), \\
&\theta_{ij} | \delta_{ij} \overset{\text{ind}}{\sim} \delta_{ij}\omega(\alpha_{i,j,1}) + (1-\delta_{ij})\omega(\alpha_{i,j,0}), \\
&\delta_{ij} \overset{\text{ind}}{\sim} \text{Bernoulli}(\pi_{ij}), \\
&\text{logit}(\pi_{ij}) = \log\left(\frac{\pi_{ij}}{1-\pi_{ij}}\right) = \boldsymbol{Z}_i^\top \boldsymbol{\xi}_j, \\
&\log(\alpha_{i,j,l}) = \boldsymbol{Z}_i^\top \boldsymbol{\eta}_{j,l}, \quad l = 0, 1.
\end{aligned}
$$

We can extend the algorithm in Section 4.4 in the main text to estimate the unknown parameters $\boldsymbol{\xi} = (\boldsymbol{\xi}_1, \ldots, \boldsymbol{\xi}_m)$ and $\boldsymbol{\eta}_l = (\boldsymbol{\eta}_{1,l}, \ldots, \boldsymbol{\eta}_{m,l})$ with $l = 0, 1$.

Remark A1 A special case of this framework allows us to perform K-means type clustering. Specifically, $\boldsymbol{Z}_i$ is a vector with the lth component equal to 1 and all the other

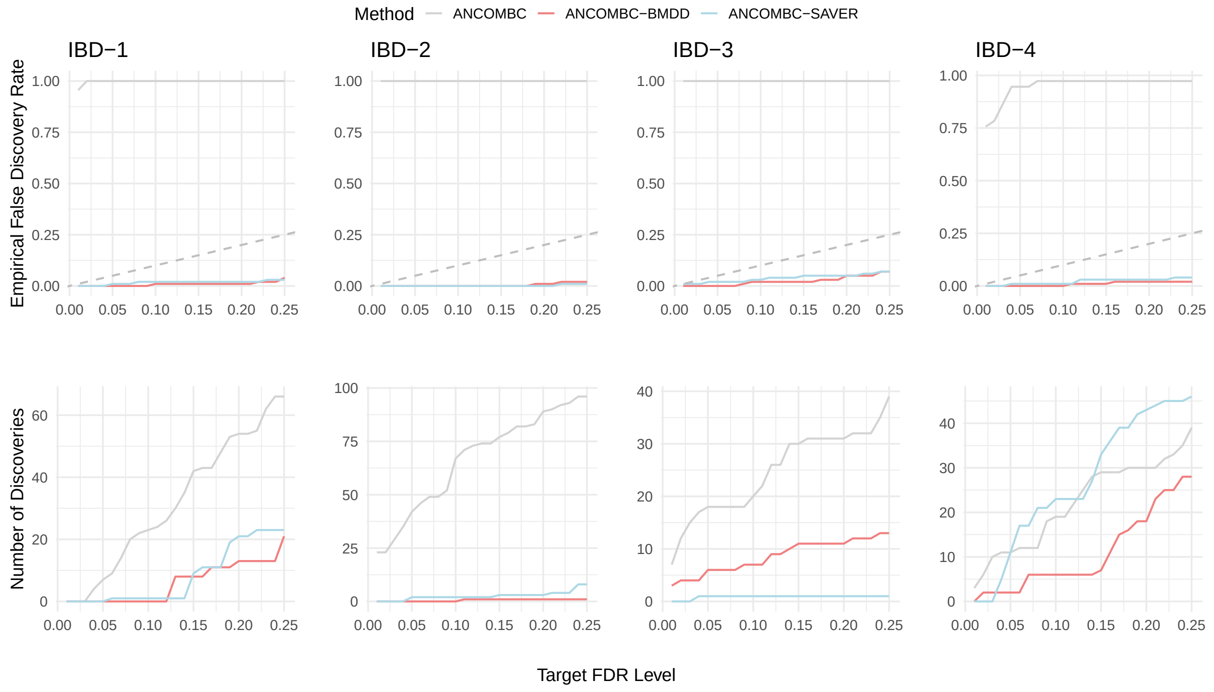

Figure A2: Performance of ANCOM-BC with BMDD on the four real datasets. (Bottom) Number of discoveries vs. target FDR level for the real datasets; (Top) Empirical FDR vs. target FDR level for the shuffled real datasets. The dashed gray line represents the diagonal. The results were averaged over 100 simulation runs.

components equal to zero if the ith subject belongs to the group l. Here the group assignment (i.e., which component of $\boldsymbol{Z}_i$ is 1) is unknown. Thus, we need to iteratively learn the group assignment together with the unknown parameters $\boldsymbol{\xi}_j$ and $\boldsymbol{\eta}_{j,l}$.

A4.2 Incorporating the phylogenetic tree information

Besides sample-level covariates, our model is also possible to accommodate the phylogeny among taxa. Our idea is to define a set of covariates (taxon-level covariates) that encode the phylogenetic information and use these covariates in the modeling. One way to define the taxon-level covariates is using the group information, e.g., the phylum of the taxa. In addition, we can compute the patristic distance between taxon i and j (denoted by d_{ij}) as the length of the shortest path linking the two taxa. Write $\mathbf{D} = (d_{ij})_{i,j=1}^m$ as the corresponding distance matrix. Then, we perform principal coordinate analysis on $\mathbf{D}$ to obtain the principal coordinates (PC) associated with the first K leading eigenvalues. These PCs can then be treated as the covariates in our structure adaptive learning procedure below.

In either case, we let $\boldsymbol{U}_j$ be the covariate derived from the phylogenetic tree for the jth taxon. We can incorporate the covariate into our model by considering

$$\text{logit}(\pi_{ij}) = \log\left(\frac{\pi_{ij}}{1-\pi_{ij}}\right) = \boldsymbol{Z}_i^\top \boldsymbol{\xi}_j + \boldsymbol{U}_j^\top \boldsymbol{\zeta}_i,$$
$$\log(\alpha_{i,j,l}) = \boldsymbol{Z}_i^\top \boldsymbol{\eta}_{j,l} + \boldsymbol{U}_j^\top \boldsymbol{\kappa}_{i,l}, \quad l = 0, 1.$$

Remark A2 The way of deriving covariates from the phylogenetic tree is far from unique. In the phylogenetic tree, each taxon is associated with a leaf node of the tree. As illustrated by Figure A3, V_j with $1 \leq j \leq 6$ denote the internal nodes with V_1 being the root node and X_j with $1 \leq j \leq 7$ are the leaf nodes corresponding to the taxa. Suppose the internal node V_j has m_j children. Then it divides the leaf nodes into $m_j + 1$ groups, where the first m_j groups correspond to the off-springs of its m_j children (including the children themselves if they are already terminal nodes), and the last group contains all the non-off-springs. For example, consider the root node V_1, which divides the seven leaf nodes into two groups, namely $\{X_1, X_2, X_3, X_4, X_5\}$ and $\{X_6, X_7\}$. In contrast, the leaf nodes form four groups with respect to the internal node V_5, i.e., $\{X_1, X_2, X_6, X_7\}$, $\{X_3\}$, $\{X_4\}$ and $\{X_5\}$. As a result, we can construct six covariates for each leaf node based on the groupings induced by each of the six internal nodes. Specifically, we let $u_{j,k}$ be a categorical variable indicating to which group (induced by the internal node V_k) X_j belongs. Generally, we can define the set of covariates for each taxon derived from the phylogenetic tree as $\mathbf{u}_j = (u_{j,1}, \ldots, u_{j,I})$ for $1 \leq j \leq m$, where I is the number of internal nodes. In practice, we may consider including internal nodes at a specific level of the tree to reduce the number of covariates.

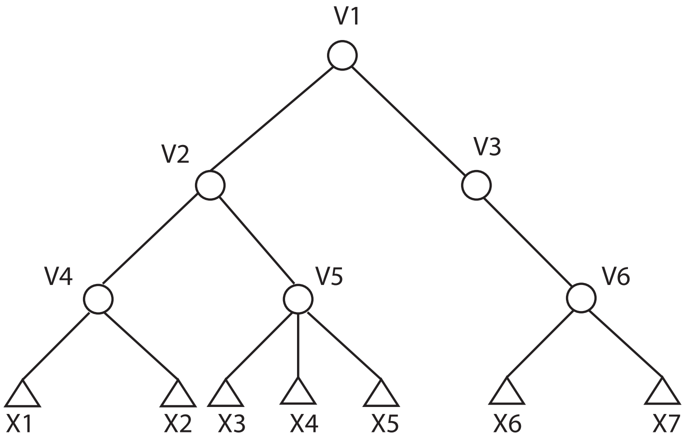

Figure A3: A simplified illustration of the phylogenetic tree. ◯ denotes internal node while △ represents leaf node.

References

Arisdakessian, C., Poirion, O., Yunits, B., Zhu, X. & Garmire, L. X. (2019), 'Deepimpute: an accurate, fast, and scalable deep neural network method to impute single-cell rna-seq data', *Genome biology* **20**(211), 1–14.

Charlson, E. S., Chen, J., Custers-Allen, R., Bittinger, K., Li, H., Sinha, R., Hwang, J., Bushman, F. D. & Collman, R. G. (2010), 'Disordered microbial communities in the upper respiratory tract of cigarette smokers', *PloS one* **5**(12), e15216.

Gevers, D., Kugathasan, S., Denson, L. A., Vázquez-Baeza, Y., Van Treuren, W., Ren, B., Schwager, E., Knights, D., Song, S. J., Yassour, M. et al. (2014), 'The treatment-naive microbiome in new-onset crohn's disease', *Cell host & microbe* **15**(3), 382–392.

Mah, C., Jayawardana, T., Leong, G., Koentgen, S., Lemberg, D., Connor, S. J., Rokkas, T., Grimm, M. C., Leach, S. T. & Hold, G. L. (2023), 'Assessing the relationship between the gut microbiota and inflammatory bowel disease therapeutics: a systematic review', *Pathogens* **12**(2), 262.

Morgan, X. C., Tickle, T. L., Sokol, H., Gevers, D., Devaney, K. L., Ward, D. V., Reyes, J. A., Shah, S. A., LeLeiko, N., Snapper, S. B. et al. (2012), 'Dysfunction of the intestinal microbiome in inflammatory bowel disease and treatment', *Genome biology* **13**(R79), 1–18.

Papa, E., Docktor, M., Smillie, C., Weber, S., Preheim, S. P., Gevers, D., Giannoukos, G., Ciulla, D., Tabbaa, D., Ingram, J. et al. (2012), 'Non-invasive mapping of the gastrointestinal microbiota identifies children with inflammatory bowel disease', *PloS one* **7**(6), e39242.

Scher, J. U., Sczesnak, A., Longman, R. S., Segata, N., Ubeda, C., Bielski, C., Rostron, T., Cerundolo, V., Pamer, E. G., Abramson, S. B. et al. (2013), 'Expansion of intestinal prevotella copri correlates with enhanced susceptibility to arthritis', *elife* **2**, e01202.

Schirmer, M., Denson, L., Vlamakis, H., Franzosa, E. A., Thomas, S., Gotman, N. M., Rufo, P., Baker, S. S., Sauer, C., Markowitz, J. et al. (2018), 'Compositional and temporal changes in the gut microbiome of pediatric ulcerative colitis patients are linked to disease course', *Cell host & microbe* **24**(4), 600–610.

Schubert, A. M., Rogers, M. A., Ring, C., Mogle, J., Petrosino, J. P., Young, V. B., Aronoff, D. M. & Schloss, P. D. (2014), 'Microbiome data distinguish patients with clostridium difficile infection and non-c. difficile-associated diarrhea from healthy controls', *MBio* **5**(3), e01021–14.

Willing, B. P., Dicksved, J., Halfvarson, J., Andersson, A. F., Lucio, M., Zheng, Z., Järnerot, G., Tysk, C., Jansson, J. K. & Engstrand, L. (2010), 'A pyrosequencing study in twins shows that gastrointestinal microbial profiles vary with inflammatory bowel disease phenotypes', *Gastroenterology* **139**(6), 1844–1854.

Wu, G. D., Chen, J., Hoffmann, C., Bittinger, K., Chen, Y.-Y., Keilbaugh, S. A., Bewtra, M., Knights, D., Walters, W. A., Knight, R. et al. (2011), 'Linking long-term dietary patterns with gut microbial enterotypes', *Science* **334**(6052), 105–108.

Yilmaz, B., Juillerat, P., Øyås, O., Ramon, C., Bravo, F. D., Franc, Y., Fournier, N., Michetti, P., Mueller, C., Geuking, M. et al. (2019), 'Microbial network disturbances in relapsing refractory crohn's disease', *Nature medicine* **25**(2), 323–336.

Zhou, H., He, K., Chen, J. & Zhang, X. (2022), 'Linda: linear models for differential abundance analysis of microbiome compositional data', *Genome biology* **23**(95), 1–23.
