# Supplementary material for: BMDD: A probabilistic framework for accurate imputation of zero-inflated microbiome sequencing data
Source: PLoS Comput Biol. 2025 Oct 24;21(10):e1013124. doi: 10.1371/journal.pcbi.1013124 (PMC12582502; doi:10.1371/journal.pcbi.1013124)
Supplement: Attachment — Submitted filename: review.pdf [file pcbi.1013124.s002.pdf]

Review

PCOMPBIOL-D-25-00901

The manuscript "BMDD: A Probabilistic Framework for Accurate Imputation of Zero-Inflated Microbiome Sequencing Data" introduces a method for imputing microbiome sequencing data. The approach aims to model the bimodal abundance distribution of taxa via a mixture of Dirichlet priors, with estimation performed through a variational EM procedure. While the core idea is promising, several substantial concerns need to be addressed to strengthen the manuscript.

Comments

- My primary concern is the novelty of the proposed bivariate Dirichlet distribution. I suggest that the authors consider the generalized Dirichlet distribution (GED), as discussed in `https://www.sciencedirect.com/science/article/pii/S0047259X12001753`. The proposed BMDD may arise as a special case of the GED. If this is the case, the authors should better motivate the BMDD in relation to the full bimodal GED. Even if BMDD offers novel aspects, its relationship to the GED must still be clarified.
- (Fig. 1C) summarizes Table A1 under setting S1. However, I find settings S2–S9 in the appendix, but no S1. Although S1 is referred to as BDMM with some parameter estimates at the end of page 9, (1) it is difficult for the reader to locate, and (2) the specific parameter values are not provided. Since settings S2–S9 are explicitly included, I recommend adding a clear definition of S1 with its exact specifications. Additionally, as stated in the legend of Table A1, the results are based on 10 Monte Carlo samples, which is not a sufficiently large number of replications.

  Furthermore, the authors should clarify how the plotted values are calculated, as they appear to summarize multiple metrics. I suggest including an explicit equation showing how the values plotted in Figure 1C are derived from Table A1.
- Following on the point of MC replicates – how many replications are used for the other simulations?
- Since the case of near-unimodality ($\alpha_{0,j} \approx \alpha_{1,j}$) is considered a relevant scenario, why is the restriction $\alpha_{0,j} = \alpha_{1,j} = \alpha_j$ not included as a competing model?
- (Fig. 3) Since SAVER is performing closesly to BDMM it should be included for comparison in computational time
- (Page 16) Why do you filter out lowly expressed taxas? Seem to go against the theme of the BDMM
- (Page 28) The authors should clearly state that the results now depend on the tightness of the bound–computational efficiency comes at a cost. Additionally, I recommend noting that this follows from Jensen's inequality, which is not particularly tight for $\log \Gamma$ over the full domain. Below is an example with Monte Carlo estimates for $X \sim 1 + \text{Gamma}(1, \text{rate})$ to illustrate this point:

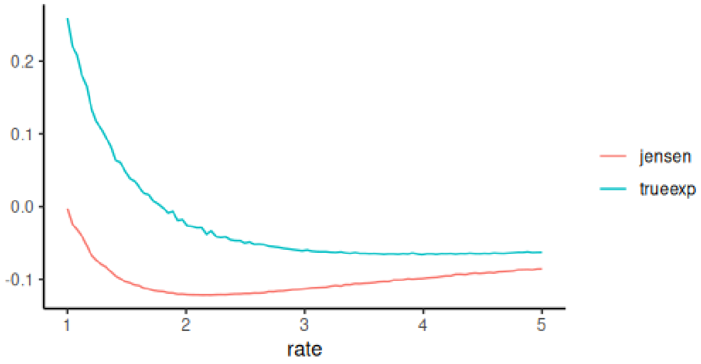

I don't think it is necessary to include simulations in the manuscript but it might be a good idea for the authors to compare with estimates from MCMC (i.e. `stan`) and compare. If there are no meaningful differences a short statement in the paper would adress the issue.

- (Suppl. Page 3) I appreciate that there's many degrees of freedom in chosing a correlation structure. Due to the complicated nature of yours, I was curious about the process of choosing it. Is it designed to be as "messy" as possible or is there any other theme behind its design?

Minor comments

Going through this manuscript, I get the distintict feeling that this manuscript has not been checked by anyone from the "outside". I find that the paper is severely lacking in organization, definition of notations, and equation numbering. While some of these issues are minor individually, collectively they result in a manuscript that is, at best, difficult to navigate for me. This list is not exhaustive, but highlights the issues I find most critical.

- The manuscript lacks consistent equation numbering. For example, in Section A1.2, you write: *we use S2–S5 to denote the settings with the four distributions*, which only implies (after close reading) that S2 = Gamma, ..., S5 = NB. This should be rephrased more clearly, e.g., "S2–S5 refer to taxa distributions (Equation X: Gamma) through (Equation Y: Negative Binomial)." Please be precise.
- In the first sentence of the introduction, the word *player* feels too vague and loose. It only gains context toward the end of the same paragraph with *Complementary to existing omics data...*. Consider rephrasing.
- The meaning of *informative prior* (page 5) is never clarified. Do you mean in the sense of being proper? Please specify exactly what you mean or consider removing the sentence.
- (Fig. 1B) You should not assume the reader understands *The blue curve is the density of the marginal beta distribution from the fit of the Dirichlet distribution.* Do you mean the marginal distribution based on the variational posterior of $\boldsymbol{X}$ in Section 4.1? Please refer explicitly to the relevant section and clarify that the marginals of Dirichlet are beta distributed.
- The sentence *Under this model, the posterior density of the true composition is computationally intractable, so we use a variational inference approach to approximate the posterior* (end of page 8) should be clarified. Intractable in what sense – analytically? While this is expanded on in Section 4.3, please either reference that section directly or elaborate here.
- The domains of variables are often undefined. For example, on page 7, it should be stated: "Let $Y_j > 0$ be generated independently from a Gamma distribution with shape parameter $\alpha_j > 0$ and scale parameter $\rho > 0$."
- Please include the code used to conduct the simulation studies in `https://github.com/zhouhj1994/BMDD/tree/master`, or refer to a separate repository in the `README.md` for replication purposes.

- What is the purpose of the first part of Section 4.1, up to *An alternative way to define BMDD is through the following hierarchical model*? This could be replaced more succinctly with: *An alternative formulation of (equation numbers) defines BMDD through the following hierarchical model* where equation numbers are referring to the first instance of BMDD.

- The section on the correlation structure of S2–S5 must be improved. For example, the sentence *taxa* $1 \sim 5$ *are simultaneously from either the larger or smaller mode, on the contrary to taxa* $6 \sim 10$*;* is unclear. First, what does $\sim$ mean here? Second, the phrase *on the contrary to taxa* $6 \sim 10$ is ambiguous–does it mean taxa 6 to 10 come from the opposite mode, or not all from the same mode? I suggest rewriting as: *Taxa 1–5 are simultaneously drawn from either the larger or smaller mode in each sample, while taxa 6–10 are drawn from the opposite mode.* This avoids the undefined $\sim$ and removes ambiguity.

- While $\boldsymbol{X}$ and $\boldsymbol{W}$ are clearly defined in Section 4.2, their estimates $\hat{\boldsymbol{X}}$ and $\hat{\boldsymbol{W}}$ appear on page 10 without prior explanation. Either define $\boldsymbol{X}$ and $\boldsymbol{W}$ earlier, before their estimates appear, or revise the structure. Do you even need to introduce $\hat{\boldsymbol{X}}$, $\hat{\boldsymbol{W}}$ at this point?

- (Page 19) Move the sentence *In all analyses, we used winsorization at quantile 0.97 to reduce the impact of potential outliers as recommended* to where you describe filtering out lowly expressed counts for coherence (or vice versa).

- (Page 24) *The goal here is to estimate...* — this reads as imprecise. "Here" is vague. Why not state directly that your parameters of interest in Equation 1 are $\boldsymbol{\alpha}$, $\boldsymbol{X}$, and $\boldsymbol{\pi}$?

- (page 25) variation frequentist estimate → variational frequentist estimate

- (page 25) Prefer $\sum_{\delta_{i1},\ldots,\delta_{im}=0}^{1} \to \sum_{j=1}^{m}\sum_{\delta_{ij}=0}^{1}$ for clarity

- (Page 25) The phrase *As seen from the derivations below ...* can be removed. It adds no value, and "below" is not clearly delimited. If you refer to specific equations, cite them explicitly.

- (Fig. 5) Add color labels to the upper-right plot.
