# Supplementary material for: BMDD: A probabilistic framework for accurate imputation of zero-inflated microbiome sequencing data
Source: PLoS Comput Biol. 2025 Oct 24;21(10):e1013124. doi: 10.1371/journal.pcbi.1013124 (PMC12582502; doi:10.1371/journal.pcbi.1013124)
Supplement: Attachment — Submitted filename: Response.pdf [file pcbi.1013124.s003.pdf]

Response Letter

We sincerely thank the two reviewers for your time and effort in providing feedback on our manuscript. Your insightful comments and valuable suggestions have greatly improved our work. We have carefully revised the manuscript in accordance with your recommendations, and all comments have been addressed.

Response to Reviewer 1

1. My primary concern is the novelty of the proposed bivariate Dirichlet distribution. I suggest that the authors consider the generalized Dirichlet distribution (GED), as discussed in https://www. sciencedirect.com/science/article/pii/S0047259X12001753. The proposed BMDD may arise as a special case of the GED. If this is the case, the authors should better motivate the BMDD in relation to the full bimodal GED. Even if BMDD offers novel aspects, its relationship to the GED must still be clarified.

Ans: Thank you for this valuable suggestion. As elaborated below, BMDD differs fundamentally from GED and is not reducible to it: GED emphasizes correlation modeling in compositional data, while BMDD provides flexible modeling of componentwise bimodality. In the GED paper, the authors introduced the random vector $\boldsymbol{Y} = (Y_1, \dots, Y_m)$ with

$$Y_j = A_j + \xi_j U, \quad j = 1, \dots, m,$$

where $A_j \sim \text{Gamma}(\alpha_j, \rho)$, $j = 1, \dots, m$, are m independent gamma random variables with shape parameters $\alpha_j > 0$ and a common scale parameter $\rho > 0$, and $U \sim \text{Gamma}(\tau, \rho)$, $\tau > 0$,

is independent of $A_1, \ldots, A_m$. Moreover, $(\xi_1, \ldots, \xi_m) \sim \text{Multinomial}(1, \boldsymbol{\pi})$ is a multinomial random vector independent of U and A_j's, and $\boldsymbol{\pi} = (\pi_1, \ldots, \pi_m)$ is such that $0 \leq \pi_j < 1$ and $\sum_{j=1}^m \pi_j = 1$. By normalizing $\boldsymbol{Y}$ with the sum of its components, we obtain the composition

$$\boldsymbol{X} = (X_1, \ldots, X_m), \text{ where } X_j = \frac{Y_j}{\sum_{k=1}^m Y_k},$$

which follows a mixture of Dirichlet distributions (referred to as GED)

$$\boldsymbol{X} \sim \sum_{j=1}^m \pi_j \text{Dirichlet}(\boldsymbol{\alpha} + \tau \boldsymbol{e}_j),$$

where $\boldsymbol{\alpha} = (\alpha_1, \ldots, \alpha_m)$ and $\boldsymbol{e}_j$ denotes the unit vector with a one in the jth position and zeros elsewhere.

In our paper, we consider a two-group gamma mixture distribution for each Y_j. Using representations consistent with the GED, we write

$$Y_j = A_j + \delta_j U_j, \quad j = 1, \ldots, m,$$

where $A_j \sim \text{Gamma}(\alpha_j, \rho)$, $U_j \sim \text{Gamma}(\tau_j, \rho)$, $\delta_j \sim \text{Bernoulli}(\pi_j)$, $j = 1, \ldots, m$, are independent, and $\alpha_j, \tau_j, \rho > 0$ and $0 \leq \pi_j < 1$. The resulting composition has the distribution

$$\boldsymbol{X} \sim \sum_{\delta_1=0}^1 \cdots \sum_{\delta_m=0}^1 \prod_{j=1}^m \pi_j^{\delta_j} (1 - \pi_j)^{1-\delta_j} \text{Dirichlet}(\boldsymbol{\alpha} + \boldsymbol{\tau} \odot \boldsymbol{\delta}),$$

where $\boldsymbol{\tau} = (\tau_1, \ldots, \tau_m)$, $\boldsymbol{\delta} = (\delta_1, \ldots, \delta_m)$, and $\odot$ denotes the element-wise product so that $\boldsymbol{\tau} \odot \boldsymbol{\delta} = (\tau_1\delta_1, \ldots, \tau_m\delta_m)$. We refer to this distribution as the bimodal Dirichlet distribution (BMDD).

Our proposed BMDD differs fundamentally from the GED in two respects: (i) Although both the GED and BMDD exhibit bimodality for each component of the composition, the underlying patterns are distinct. In the BMDD, the two modes (α_j and $\alpha_j + \tau_j$) can vary arbitrarily across components. In contrast, in the GED the modes are mutually exclusive: exactly one component follows the second mode ($\alpha_j + \tau$), while all others must follow the first mode ($\alpha_k, k \neq j$). Moreover, the difference between the two modes is identical for all components, namely τ. These constraints in the GED are not realistic for modeling the

bimodal characteristics of compositional components, as it is unreasonable to assume that only a single component can arise from the larger mode. (ii) BMDD has more parameters than GED: $2m$ parameters for the GED, i.e, $\alpha_1, \ldots, \alpha_m$, $\pi_1, \ldots, \pi_m$ with the constraint that they sum to 1, plus τ; and $3m$ for the BMDD, i.e, $\alpha_1, \ldots, \alpha_m$, $\tau_1, \ldots, \tau_m$ and $\pi_1, \ldots, \pi_m$. BMDD defines a substantially more complex distribution and is therefore more expressive for modeling data: The GED is a mixture of m Dirichlet distributions, whereas the BMDD is a mixture of 2^m Dirichlet distributions.

The GED framework provides valuable insights. While the BMDD assumes independence among the Y_j's, the GED employs a multinomial random vector $(\xi_1, \ldots, \xi_m)$, which induces correlations between the Y_j's. Since taxa abundances may be correlated in practice, extending the BMDD to incorporate such dependencies using ideas from the GED could be a promising avenue for future research.

We clarified their distinction in the Discussion section. See revised text in red.

2. (Fig. 1C) summarizes Table A1 under setting S1. However, I find settings S2–S9 in the appendix, but no S1. Although S1 is referred to as BMDD with some parameter estimates at the end of page 9, (1) it is difficult for the reader to locate, and (2) the specific parameter values are not provided. Since settings S2–S9 are explicitly included, I recommend adding a clear definition of S1 with its exact specifications. Additionally, as stated in the legend of Table A1, the results are based on 10 Monte Carlo samples, which is not a sufficiently large number of replications.

Furthermore, the authors should clarify how the plotted values are calculated, as they appear to summarize multiple metrics. I suggest including an explicit equation showing how the values plotted in Figure 1C are derived from Table A1.

Ans: Thanks for your helpful suggestion to enhance the clarity of the paper. (i) We have added the details of the definition of S1 in Section A2.1 of the Supplementary Materials and referenced it in the main text. (ii) Table A1 summarizes the results of different methods in terms of the divergence between their estimated composition and the true composition.

As specified in Section A2.2 of the Supplementary Materials, these metrics are computed by averaging over the number of taxa ($m = 100$ in our simulations) or the sample size ($n = 80$). Consequently, the values of these metrics show little variability across 10 repeated runs, as indicated by the narrow standard errors reported in Table A1. Therefore, we performed only 10 runs, which are sufficient to illustrate the performance of the different methods. (iii) We have added the formulation of how the plotted values in Figure 1c are derived from those in Table A1 to the caption of Figure 1.

3. Following on the point of MC replicates – how many replications are used for the other simulations?

Ans: For simulation settings S2–S9, we also used 10 replications, which are sufficient to illustrate our point for the reasons provided in our response to your previous comment. For the simulations and real data analyses in differential abundance analysis, we used 500 and 100 replications, respectively, as indicated in the caption of Figure 4.

4. Since the case of near-unimodality ($\alpha_{j,0} \approx \alpha_{j,1}$) is considered a relevant scenario, why is the restriction $\alpha_{j,0} = \alpha_{j,1} = \alpha_j$ not included as a competing model?

Ans: For the case where $\alpha_{j,0} = \alpha_{j,1} = \alpha_j$, the competing model is a standard Dirichlet-multinomial (DM) distribution. Among our competing methods, we considered the Dirichlet multinomial mixture distribution (DMM), and DM is a special case of DMM with a single Dirichlet component. In our comparison, we set the number of Dirichlet mixtures to 10 in our studies. This specification is anticipated to yield better performance than a single Dirichlet component. Table R1 (provided in this response letter) confirms this intuition: DMM with 10 Dirichlet components consistently outperforms DMM with a single Dirichlet distribution (DM) across all distance metrics.

5. (Fig. 3) Since SAVER is performing closely to BMDD it should be included for comparison in computational time.

Ans: Thanks for this comment. SAVER is indeed more computationally efficient than BMDD: it requires only a few minutes (Figure R1) to handle a large number of taxa (e.g.,

Table R1: Performance comparison of BMDD, BMDD with Monte Carlo (MC) procedures (see response to comment 7), DMM with 10 Dirichlet components, and DMM with a single Dirichlet distribution for true composition estimation under setting S1. Results for BMDD and DMM (10 components) are drawn from Table A1 of the Supplementary Materials, and those for BMDD (MC) and DMM (1 component) are newly provided here.

	BMDD	BMDD (MC)	DMM (10 components)	DMM (1 component)
Mean squared error	5.25e-05(3.26e-06)	1.02e-04(9.36e-06)	1.85e-04(8.59e-06)	2.86e-04(1.64e-05)
Sample-wise distance	0.0569(0.0014)	0.0761(0.0032)	0.1329(0.0032)	0.1661(0.0047)
Taxon-wise distance	0.0502(0.0012)	0.0616(0.0015)	0.0994(0.0030)	0.1086(0.0028)
Shannon's index	0.0432(0.0029)	0.3115(0.0313)	0.3678(0.0287)	0.7950(0.0550)
Simpson's index	1.10e-04(1.75e-05)	7.82e-04(1.47e-04)	5.49e-04(3.78e-05)	0.0019(3.16e-04)
Bray-Curtis dissimilarity	0.1551(0.0035)	0.2064(0.0056)	0.3848(0.0116)	0.4567(0.0100)
Kullback–Leibler divergence	0.1668(0.0066)	0.2558(0.0125)	0.5747(0.0228)	0.6738(0.0241)
Jensen–Shannon divergence	0.0398(0.0016)	0.0642(0.0029)	0.1412(0.0055)	0.1750(0.0056)
Hellinger distance	0.1784(0.0036)	0.2255(0.0052)	0.4021(0.0081)	0.4521(0.0074)
Gini coefficient	0.0091(9.49e-04)	0.0279(0.0021)	0.2175(0.0108)	0.5011(0.0057)
(Mean, Standard deviation)	5.61e-06(5.47e-07)	2.82e-05(3.85e-06)	7.55e-05(6.84e-06)	2.89e-04(1.66e-05)
Coefficient of variation	0.0912(0.0090)	0.4229(0.0344)	1.6840(0.0717)	3.1253(0.0556)
Kolmogorov–Smirnov distance	0.2697(0.0054)	0.3165(0.0082)	0.5632(0.0102)	0.7461(0.0048)
Wasserstein distance	0.1308(0.0044)	0.2392(0.0099)	0.4463(0.0163)	0.7308(0.0161)
Pairwise taxon-to-taxon correlations	0.0060(3.80e-04)	0.0147(0.0023)	0.1462(0.0111)	1.0208(0.0013)

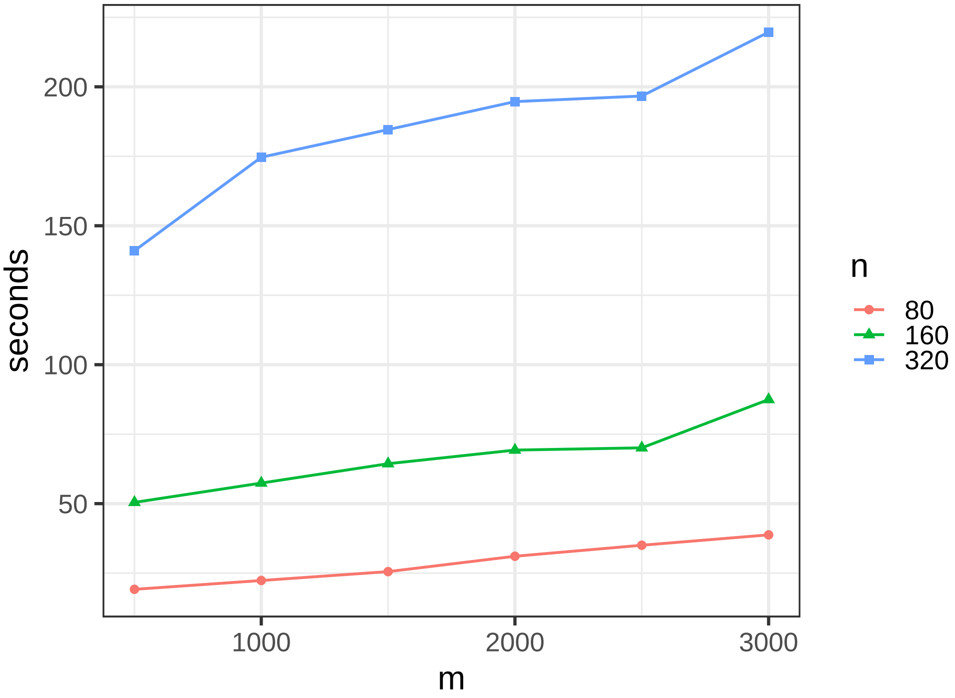

Figure R1: Execution time of the SAVER algorithm (R version 4.3.1 (2023-06-16); Platform: aarch64-apple-darwin20; CPU: Apple M2 Max; Memory: 32 GB). The y-axis represents the time (in seconds) that the SAVER algorithm needs to complete the computations.

3000), whereas BMDD may take several hours (Figure 3 of the main text). The pronounced difference in computational efficiency between the two methods arises from their fundamentally different modeling frameworks. SAVER models each count using a Poisson distribution with a gamma prior on the mean. The hyperparameters in the prior are empirically estimated by fitting linear regressions between each gene and other genes in the same cell, and the posterior can be computed efficiently due to Poisson–gamma conjugacy. In contrast, BMDD is a mixture of 2^m Dirichlet distributions, where m is the number of taxa. While this complexity makes BMDD highly expressive, it comes at the cost of computational efficiency. The model is solved using a variational EM algorithm, involving both EM iterations and coordinate ascent iterations within the E-step. As our primary goal is to provide accurate imputation for microbiome data and to facilitate downstream analyses, we allow some compromise on computational efficiency. Our simulation studies for true composition es-

timation indicate that SAVER performs reasonably well (Table A1 of the Supplementary Materials, Figures 1c and 2 of the main text). However, BMDD consistently outperforms SAVER across various settings, including both parametric models with correlation structure and nonparametric models.

Another serious problem of SAVER is that it could leak information. As it borrows information across taxa, differential signals could leak to non-differential taxa. In our differential abundance studies, using posterior samples from SAVER leads to severe FDR inflation (Figures 4a and 5 of the main text), whereas posterior samples from BMDD improve the robustness of the original differential abundance methods.

6. (Page 16) Why do you filter out lowly expressed taxa? Seem to go against the theme of the BMDD.

Ans: We assume you were referring to page 19. Filtering out low-prevalence taxa is a common preprocessing step in microbiome data analysis (Chen et al. 2018, Hu et al. 2022, Zhou et al. 2022). Because low-prevalence taxa are information-poor, their inclusion can introduce noise, increase computational demands, and weaken statistical power. This practice is motivated by several considerations: (i) Reducing noise and improving statistical power. Taxa with extremely low counts could reflect background noise due to contamination or sequencing error. These extremely rare taxa have virtually no statistical power. Including these taxa increases the multiple testing burden and reduces the power to detect true signals. (ii) Enhancing computational efficiency. Microbiome datasets often contain thousands of taxa, and removing very low-count taxa reduces the scale of the data and accelerates model fitting. (iii) Increasing the computational stability. Because these rare taxa carry little information, their imputations are unreliable, and including them can sometimes introduce computational problems.

7. (Page 28) The authors should clearly state that the results now depend on the tightness of the bound– computational efficiency comes at a cost. Additionally, I recommend noting that this follows from Jensen's inequality, which is not particularly tight for $\log \Gamma$ over the full domain. Below is an example with Monte Carlo estimates for $X \sim 1 + \mathrm{Gamma}(1, \mathrm{rate})$

to illustrate this point:

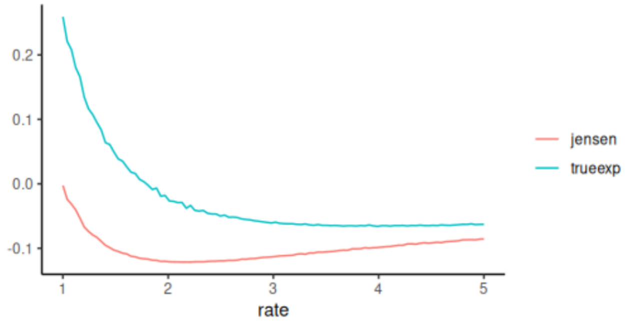

I don't think it is necessary to include simulations in the manuscript but it might be a good idea for the authors to compare with estimates from MCMC (i.e. `stan`) and compare. If there are no meaningful differences a short statement in the paper would address the issue.

Ans: This is an insightful comment. Following your suggestion, we implemented BMDD using Monte Carlo estimates instead of the approximation based on Jensen's inequality. The results are presented in the second column of Table R1 (BMDD(MC)). Interestingly, the Monte Carlo version of BMDD performed worse than our proposed approach using the Jensen-based lower bound. We suspect that this counterintuitive result arises because, with Monte Carlo estimates, the dependence of the objective function on the parameters is stochastic and lacks a closed-form expression, which introduces instability into the optimization. In contrast, with the lower bound, the dependence on the parameters is explicit and deterministic, leading to a more stable and standard optimization procedure. We have added discussions in Section 4.4.

8. (Suppl. Page 3) I appreciate that there are many degrees of freedom in choosing a correlation structure. Due to the complicated nature of yours, I was curious about the process of choosing it. Is it designed to be as "messy" as possible, or is there any other theme behind its design?

Ans: Yes, our intention was to construct the correlation structure to be as intricate as possible in order to better mimic the complexity of real-world data. To this end, we incor-

porated diverse patterns of association, reflecting that some taxa are mutualistic (positively correlated), while others are competitive (negatively correlated).

9. Minor comments

Going through this manuscript, I get the distinct feeling that this manuscript has not been checked by anyone from the "outside". I find that the paper is severely lacking in organization, definition of notations, and equation numbering. While some of these issues are minor individually, collectively they result in a manuscript that is, at best, difficult to navigate for me. This list is not exhaustive, but highlights the issues I find most critical.

- The manuscript lacks consistent equation numbering. For example, in Section A1.2, you write: *we use S2–S5 to denote the settings with the four distributions*, which only implies (after close reading) that S2 = Gamma, ..., S5 = NB. This should be rephrased more clearly, e.g., "S2–S5 refer to taxa distributions (Equation X: Gamma) through (Equation Y: Negative Binomial)." Please be precise.

- In the first sentence of the introduction, the word *player* feels too vague and loose. It only gains context toward the end of the same paragraph with *Complementary to existing omics data....* Consider rephrasing.

- The meaning of *informative prior* (page 5) is never clarified. Do you mean in the sense of being proper? Please specify exactly what you mean or consider removing the sentence.

- (Fig. 1B) You should not assume the reader understands *The blue curve is the density of the marginal beta distribution from the fit of the Dirichlet distribution.* Do you mean the marginal distribution based on the variational posterior of $\boldsymbol{X}$ in Section 4.1? Please refer explicitly to the relevant section and clarify that the marginals of the Dirichlet are beta distributed.

- The sentence *Under this model, the posterior density of the true composition is computationally intractable, so we use a variational inference approach to approximate the*

posterior (end of page 8) should be clarified. Intractable in what sense – analytically? While this is expanded on in Section 4.3, please either reference that section directly or elaborate here.

- The domains of variables are often undefined. For example, on page 7, it should be stated: "Let $Y_j > 0$ be generated independently from a Gamma distribution with shape parameter $\alpha_j > 0$ and scale parameter $\rho > 0$."

- Please include the code used to conduct the simulation studies in `https://github.com /zhouhj1994/ BMDD/tree/master`, or refer to a separate repository in the `README.md` for replication purposes.

- What is the purpose of the first part of Section 4.1, up to *An alternative way to define BMDD is through the following hierarchical model*? This could be replaced more succinctly with: *An alternative formulation of (equation numbers) defines BMDD through the following hierarchical model* where equation numbers are referring to the first instance of BMDD.

- The section on the correlation structure of S2–S5 must be improved. For example, the sentence *taxa* $1 \sim 5$ *are simultaneously from either the larger or smaller mode, on the contrary to taxa* $6 \sim 10$*;* is unclear. First, what does $\sim$ mean here? Second, the phrase *on the contrary to taxa* $6 \sim 10$ is ambiguous–does it mean taxa 6 to 10 come from the opposite mode, or not all from the same mode? I suggest rewriting as: *Taxa 1–5 are simultaneously drawn from either the larger or smaller mode in each sample, while taxa 6–10 are drawn from the opposite mode.* This avoids the undefined $\sim$ and removes ambiguity.

- While $\boldsymbol{X}$ and $\boldsymbol{W}$ are clearly defined in Section 4.2, their estimates $\widehat{\boldsymbol{X}}$ and $\widehat{\boldsymbol{W}}$ appear on page 10 without prior explanation. Either define $\boldsymbol{X}$ and $\boldsymbol{W}$ earlier, before their estimates appear, or revise the structure. Do you even need to introduce $\widehat{\boldsymbol{X}}$, $\widehat{\boldsymbol{W}}$ at this point?

- (Page 19) Move the sentence *In all analyses, we used winsorization at quantile 0.97 to*

reduce the impact of potential outliers as recommended to where you describe filtering out lowly expressed counts for coherence (or vice versa).

- (Page 24) *The goal here is to estimate...* — this reads as imprecise. "Here" is vague. Why not state directly that your parameters of interest in Equation 1 are $\boldsymbol{\alpha}$, $\boldsymbol{X}$, and $\boldsymbol{\pi}$?
- (page 25) variation frequentist estimate → variational frequentist estimate
- (page 25) Prefer $\sum_{\delta_{i1},\ldots,\delta_{im}=0}^{1} \to \sum_{j=1}^{m}\sum_{\delta_{ij}=0}^{1}$ for clarity
- (Page 25) The phrase *As seen from the derivations below...* can be removed. It adds no value, and "below" is not clearly delimited. If you refer to specific equations, cite them explicitly.
- (Fig. 5) Add color labels to the upper-right plot.

Ans: We really appreciate your careful reading and helpful suggestions, which have significantly improved the readability of the manuscript. We have now fixed these issues in the revision. The full source code for reproducing the simulation results has been deposited at https://github.com/zhouhj1994/BMDD-manuscript-sourcecode. Please see the text highlighted in red.

Response to Reviewer 2

1. How do you ensure identifiability between the two Gamma components in the mixture model? Especially for taxa with very sparse signals, the difference between the two α parameters ($\alpha_{j,0}$ and $\alpha_{j,1}$) may be subtle. Could different parameter combinations produce similar outputs?

Ans: Thanks for this insightful comment. Finite mixtures of Gamma distributions are generally identifiable up to label switching. When the parameters $(\alpha_{j,0}, \alpha_{j,1})$ are close, numerical

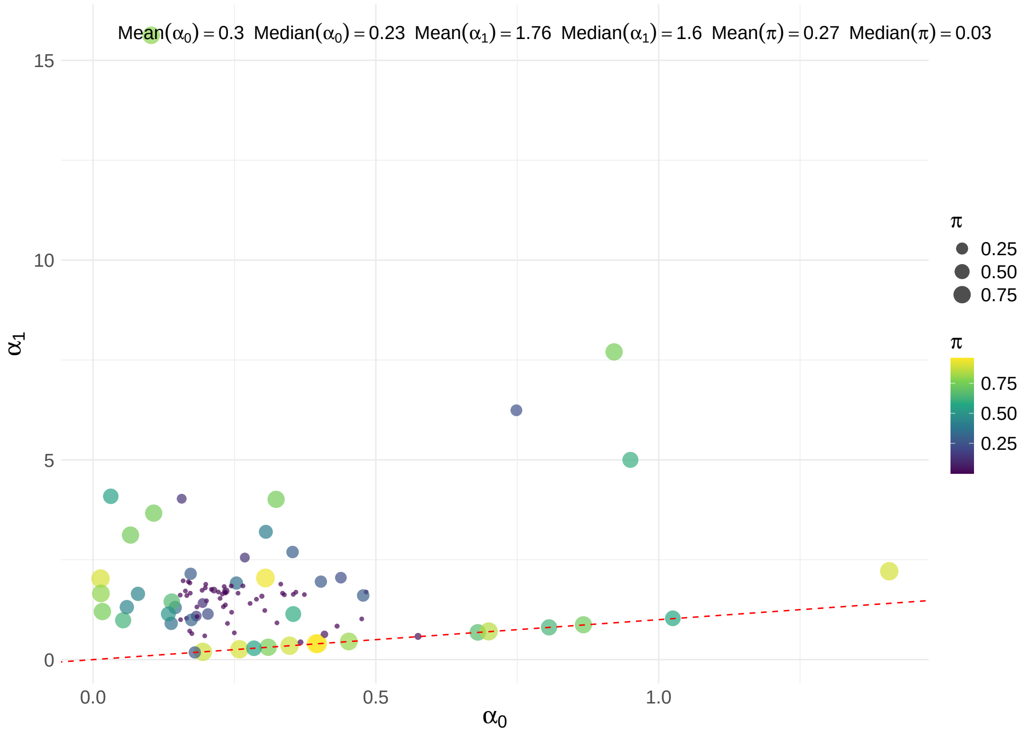

Figure R2: Parameter estimates from the BMDD fit to the COMBO dataset. The dashed red line represents the diagonal where $\alpha_0 = \alpha_1$.

instability may arise in estimation, and these parameters can not be estimated accurately. Different estimates of $(\alpha_{j,0}, \alpha_{j,1}, \pi_j)$ could explain the data equally well with very close likelihood when $(\alpha_{j,0}, \alpha_{j,1})$ are close. To the extreme case where the data follows a strictly unimodal Dirichlet distribution $(\alpha_{j,0} = \alpha_{j,1})$, the parameters are not identifiable. However, as our major aim is to accurately estimate the underlying true composition, not the parameters themselves, over-parameterization is more beneficial than under-parameterization.

Let us consider an example demonstrating how our proposed algorithm behaves when $\alpha_{j,0}$ and $\alpha_{j,1}$ are nearly equal. In our simulations under setting S1, we fitted the BMDD to the COMBO dataset (Wu et al. 2011) and obtained estimates of the parameters $\alpha_{j,0}, \alpha_{j,1}, \pi_j$ for $j = 1, \ldots, m$ ($m = 100$ in our study), as shown in Figure R2. We observe that some $\alpha_{j,0}$ and $\alpha_{j,1}$ values are indeed close to each other (near the diagonal line). We use these parameter values to generate BMDD samples and apply our algorithm to estimate them.

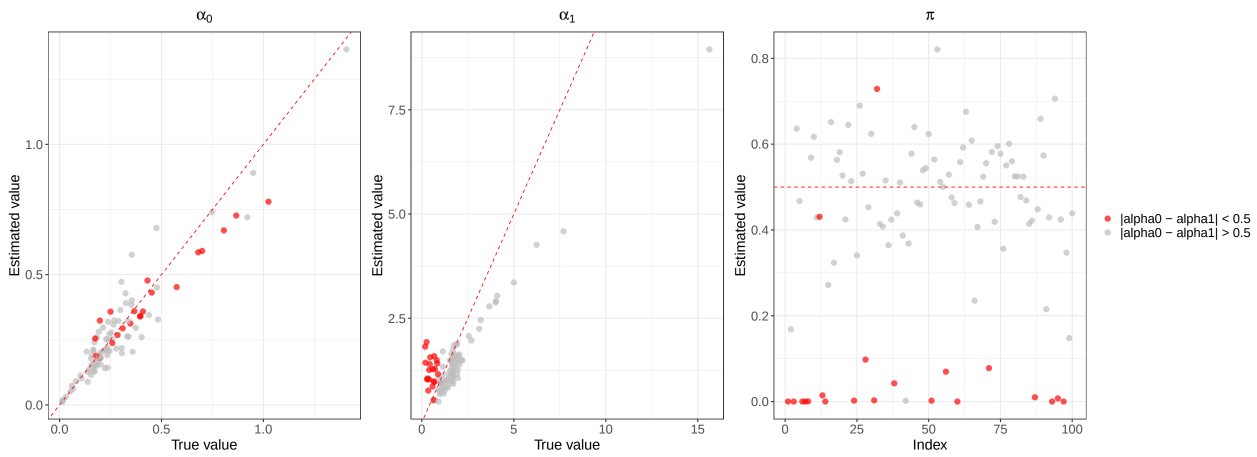

Figure R3: Parameter estimates versus true values. In the first two plots, the dashed red line represents the diagonal, while in the third plot it indicates the horizontal line at 0.5.

Figure R3 shows the estimation results. The estimates for $\alpha_{j,1}$ are generally accurate, while some estimates for $\alpha_{j,1}$ are less precise. Upon inspection, these less accurate estimates correspond to cases where $\alpha_{j,0}$ and $\alpha_{j,1}$ are close ($\alpha_{j,1} - \alpha_{j,0} < 0.5$, highlighted in red), and the corresponding estimates for π_j are near zero. Thus, these deviations do not compromise the overall fit.

2. How stable and biologically interpretable is the π_j parameter across datasets? Since π_j reflects the probability of the second (non-zero) mode, do you observe consistency in π_j for known opportunistic taxa or context-dependent microbes?

Ans: We compare the genus-level estimates of π_j for the COMBO dataset, derived from a cross-sectional study of dietary effects on stool microbiome composition (Wu et al. 2011), and stool samples from the Human Microbiome Project (HMP) dataset (Schiffer et al. 2019). Figure R4 shows that the estimated values of π_j are largely consistent across the two datasets, with most genera having a π_j close to zero (dominated by the low-abundance mode). However, some genera, particularly *Parabacteroides*, *Eubacterium*, and *Sutterella*, exhibit distinct distribution patterns, and their π_j estimates could be very different. We speculate that the discrepancy may be due to the difference in population characteristics, sample preparation, and sequencing methods.

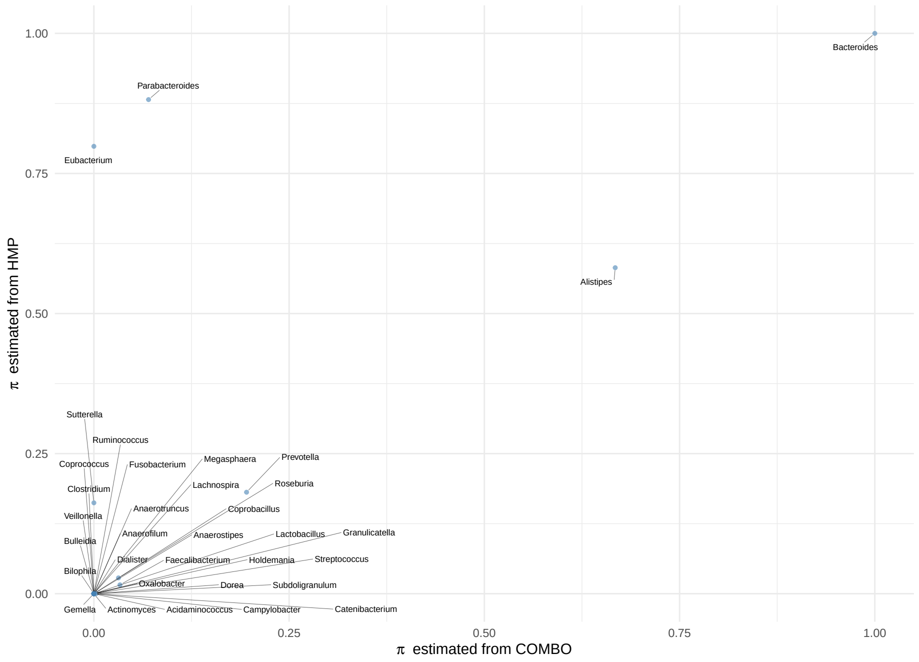

Figure R4: Genus-level estimates of π_j from the COMBO and HMP datasets.

3. Though you state that the final composition is independent of ρ, does the choice of ρ affect inference stability or convergence in practice?

Ans: The composition generated by the BMDD is not affected by the choice of ρ. The parameterρ influences the unobserved absolute abundances. In our simulations, we derived the true composition by normalizing the absolute abundances generated from the two-group gamma mixture distributions. To examine whether different values of ρ might introduce numerical issues that affect inference stability, we generated data under varying ρ. The results, presented in Table R2, indicate that the inference performance of BMDD is not affected by the choice of ρ.

4. Have you performed posterior predictive checks to evaluate whether BMDD accurately reproduces multimodal structures in held-out taxa? A visual or statistical PPC would build confidence in model fit and flexibility.

Ans: Thanks for this suggestion. We have now performed a posterior predictive check

Table R2: Performance of BMDD under setting S1 with different scale parameter of the gamma distribution (ρ). The baseline setting used in the paper corresponds to $\rho = 1$, with results reported in Table A1 of the Supplementary Materials. The results for BMDD with $\rho = 0.1$ and $\rho = 0.01$ are newly provided here.

	BMDD	BMDD ($\rho = 0.1$)	BMDD ($\rho = 0.01$)
Mean squared error	5.25e-05(3.26e-06)	5.28e-05(3.16e-06)	5.03e-05(2.15e-06)
Sample-wise distance	0.0569(0.0014)	0.0570(0.0013)	0.0564(0.0011)
Taxon-wise distance	0.0502(0.0012)	0.0503(0.0012)	0.0490(0.0014)
Shannon's index	0.0432(0.0029)	0.0443(0.0044)	0.0434(0.0048)
Simpson's index	1.10e-04(1.75e-05)	1.11e-04(1.77e-05)	9.66e-05(1.39e-05)
Bray-Curtis dissimilarity	0.1551(0.0035)	0.1552(0.0035)	0.1529(0.0041)
Kullback–Leibler divergence	0.1668(0.0066)	0.1681(0.0067)	0.1645(0.0063)
Jensen–Shannon divergence	0.0398(0.0016)	0.0399(0.0015)	0.0389(0.0013)
Hellinger distance	0.1784(0.0036)	0.1785(0.0035)	0.1768(0.0029)
Gini coefficient	0.0091(9.49e-04)	0.0091(9.85e-04)	0.0089(4.69e-04)
(Mean, Standard deviation)	5.61e-06(5.47e-07)	5.59e-06(4.79e-07)	4.84e-06(3.11e-07)
Coefficient of variation	0.0912(0.0090)	0.0882(0.0085)	0.0830(0.0069)
Kolmogorov–Smirnov distance	0.2697(0.0054)	0.2695(0.0054)	0.2755(0.0050)
Wasserstein distance	0.1308(0.0044)	0.1307(0.0041)	0.1289(0.0033)
Pairwise taxon-to-taxon correlation	0.0060(3.80e-04)	0.0061(3.96e-04)	0.0058(3.03e-04)

(PPC) to see if BMDD accurately reproduces the observed distribution. Using data from the American Gut Project as an example, we generated a count matrix by drawing samples from multinomial distributions parameterized with posterior samples of the compositions from BMDD. As shown in Figure A1 of the Supplementary Materials for the four example taxa in Figure 1a, the generated data closely resemble the distribution of the observed data. We have added the PPC result in Section 2.1 of the main text.

5. Could the BMDD framework be extended to incorporate temporal or longitudinal structure? For microbiome studies with repeated measurements (e.g., interventions), accounting for temporal dynamics would be highly valuable.

Ans: Thank you for this valuable suggestion. Our BMDD framework can be extended to accommodate data with repeated measurements or longitudinal structures. In Section A4.1 of the Supplementary Materials, we introduce a mixture regression framework that incorporates sample covariates. As a further step, mixed-effects regression can be applied to such data. However, this extension would considerably increase the model complexity and require more sophisticated algorithms. We believe this represents a promising direction for future research. We added some discussion regarding this extension. See the highlighted text in the Discussion section.

6. Is the bimodal assumption flexible enough for cases with more than two ecological states? Some taxa may have trimodal or multimodal behavior depending on niche availability or host status.

Ans: Thanks for your insightful comment. Indeed, some taxa may exhibit trimodal or more complex multimodal behaviors depending on ecological niches or host conditions. Our current BMDD framework focuses on the bimodal case, which we believe captures a wide range of realistic scenarios in microbiome data where taxa tend to cluster into “rare” and “abundant” states. Nevertheless, our framework is flexible and can be extended to mixtures of more than two Dirichlet components (for example, by allowing δ_{ij} in formula (1) of the main text to follow a multinomial distribution instead of a Bernoulli distribution). Such extensions would enable modeling taxa with multiple ecological states, albeit with increased

model complexity and parameter estimation challenges. We see this as a promising direction for future research.

7. Can you connect the inferred bimodality to known ecological transitions (e.g., bloom dynamics, keystone switching)? Showing such links would strengthen the biological impact of your model.

Ans: We appreciate the reviewer's thoughtful suggestion to link inferred bimodality to specific ecological transitions (e.g., bloom dynamics, keystone switching) and strongly agree that doing so would substantially enhance the biological impact of our work. However, rigorous attribution of bimodal signals to specific ecological mechanisms would require substantial ecological expertise, appropriate datasets, and targeted analyses beyond the scope of this paper and we prefer to avoid speculative interpretations due to our limited experience in ecological research. We will pursue this direction in future work. We added some discussions about the potential downstream application of our method.

8. these are the suggestions that the paper could benefit from:

- Discuss how inferred π_j values could be used to stratify taxa (e.g., core vs. transient microbes).
- While Figure 1b shows the generative process, a higher-level conceptual diagram summarizing assumptions and inference flow (akin to a graphical model) would help non-statistical readers.
- Especially for real data, show how the model captures bimodal or zero-inflated features—e.g., overlaid histograms or kernel density plots of observed vs. simulated abundance for selected taxa.
- Explicitly discuss:

  When the bimodal assumption may fail

  Whether the variational posterior converges reliably across taxa

  How model performance scales with increasing taxa or deeper sequencing

- It's not clear if code for BMDD is publicly available. Publishing the pipeline (e.g., as an R or Python package) would vastly improve its adoption.
- The multiple imputation framework is appealing. But it's worth clarifying:

  How many posterior samples are recommended in practice?

  Is Rubin's rule applicable for any DA method, or just linear models?

Ans: Thanks for these helpful suggestions, which significantly improved the clarity of the work and the depth of the discussion.

(i) We have added some discussion about the use of π_j for stratifying taxa. See the highlighted text in Discussion.

(ii) We have included a schematic diagram in Section 4.2 following the introduction of our hierarchical model.

(iii) See our reply to your comment 4.

(iv) Following the suggestions, we have explicitly discussed the following points. See highlighted texts in Discussion.

- Similar to the response to comment 1, even if the data are unimodally distributed, our model could still fit the data better (Figure 1a). However, if the data is marginally beta distributed, our model is over-parameterized, but it could still generate accurate estimates of the underlying compositions. In Section 2 of the main text, we evaluated the robustness of our method under both model misspecification and non-parametric settings that mimic real-world data (without the biomodal assumption).
- To illustrate the convergence of our variational algorithm, we tracked the average posterior mean across samples at each iteration under different initializations. Figure R5 shows the results for four example taxa (those used in Figure 1a). We observe that the average posterior means converge to similar values across different initializations

for each taxon, although a formal theoretical guarantee of convergence requires further investigation.

- Table R3 shows the performance of BMDD under varying sequencing depths and numbers of taxa. As the sequencing depth increases, the count matrix contains fewer zeros, is less noisy, and provides more information, leading to more accurate recovery of the true composition. Consequently, the divergences between the true and estimated compositions decrease across all metrics as sequencing depth increases. Conversely, increasing the number of taxa while keeping the sequencing depth fixed leads to a sparser count matrix, which reduces estimation efficiency. We observe that the estimation performance worsens for Preservation of sample-wise properties (metrics 4–9) and Preservation of taxon-wise properties (metrics 10–15). Interestingly, the performance of General similarity measures between two composition matrices (metrics 1–3) improves as the number of taxa increases. This is expected because as m grows, each individual composition value becomes smaller, resulting in lower squared losses.

(v) BMDD is implemented as the `bmdd` function in the R package *BMDD* and is available on GitHub at https://github.com/zhouhj1994/BMDD.

(vi) We have added discussions on the choice of posterior sample size and the application of Rubin's rule in the Discussion section.

References

Chen, J., King, E., Deek, R., Wei, Z., Yu, Y., Grill, D. & Ballman, K. (2018), 'An omnibus test for differential distribution analysis of microbiome sequencing data', *Bioinformatics* **34**(4), 643–651.

Hu, Y., Satten, G. A. & Hu, Y.-J. (2022), 'Locom: A logistic regression model for testing differential abundance in compositional microbiome data with false discovery rate control', *Proceedings of the National of Sciences* **119**(30), e2122788119.

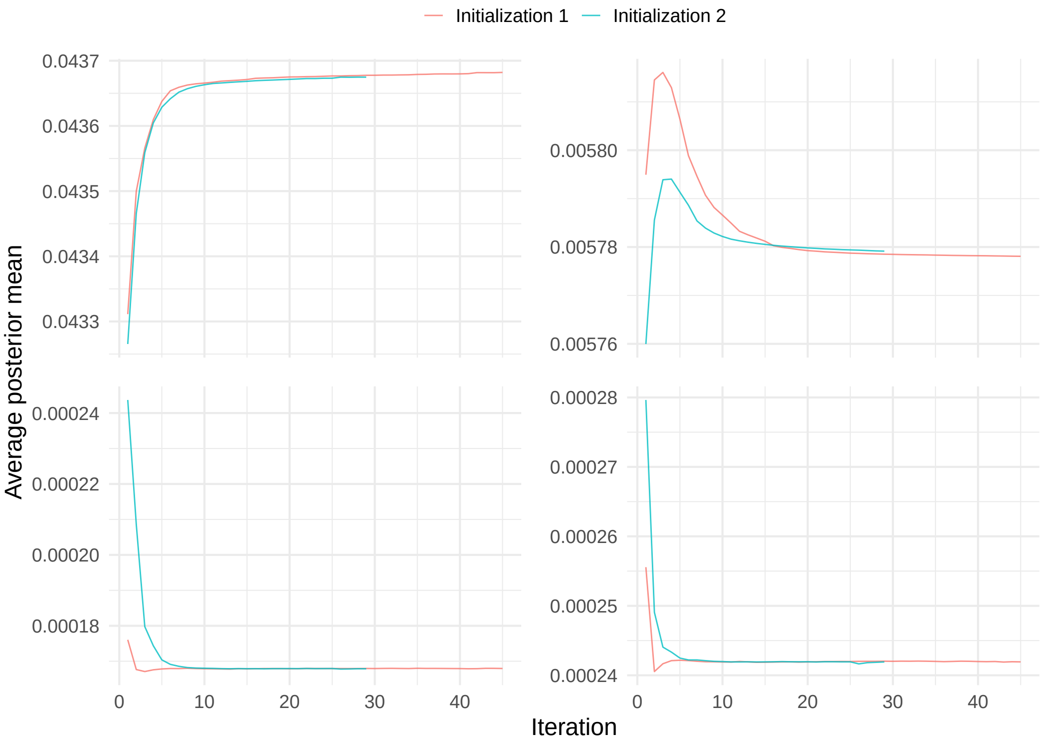

Figure R5: Convergence of the average posterior mean across samples under different initializations for the four example taxa from the American Gut Project.

Table R3: Performance of BMDD under setting S1 with different sequencing depths (d) and number of taxa m. The baseline setting used in the paper corresponds to $d = 1000$ and $m = 100$, with results reported in Table A1 of the Supplementary Materials. Four new settings are included here: BMDD with $d = 5000$ and $d = 50000$ (both with $m = 100$), and BMDD with $m = 200$ and $m = 500$ (both with $d = 1000$).

	BMDD	BMDD ($d = 5000$)	BMDD ($d = 50000$)	BMDD ($m = 200$)	BMDD ($m = 500$)
Mean squared error	5.25e-05(3.26e-06)	2.42e-05(2.20e-06)	8.02e-06(1.06e-06)	1.96e-05(1.17e-06)	5.07e-06(5.45e-07)
Sample-wise distance	0.0569(0.0014)	0.0332(0.0014)	0.0149(6.64e-04)	0.0533(0.0016)	0.0450(0.0022)
Taxon-wise distance	0.0502(0.0012)	0.0332(0.0012)	0.0185(0.0012)	0.0304(7.45e-04)	0.0146(5.20e-04)
Shannon's index	0.0432(0.0029)	0.0163(0.0022)	0.0056(9.38e-04)	0.1087(0.0158)	0.2478(0.0585)
Simpson's index	1.10e-04(1.75e-05)	5.00e-05(1.07e-05)	1.87e-05(4.32e-06)	6.04e-05(1.46e-05)	2.40e-05(5.48e-06)
Bray-Curtis dissimilarity	0.1551(0.0035)	0.0893(0.0032)	0.0401(0.0019)	0.2050(0.0052)	0.2671(0.0126)
Kullback–Leibler divergence	0.1668(0.0066)	0.0787(0.0049)	0.0279(0.0031)	0.2359(0.0097)	0.3389(0.0276)
Jensen–Shannon divergence	0.0398(0.0016)	0.0186(0.0012)	0.0065(7.25e-04)	0.0577(0.0024)	0.0836(0.0065)
Hellinger distance	0.1784(0.0036)	0.1059(0.0035)	0.0486(0.0019)	0.2281(0.0054)	0.2880(0.0109)
Gini coefficient	0.0091(9.49e-04)	0.0020(3.11e-04)	2.93e-04(4.01e-05)	0.0189(0.0017)	0.0444(0.0072)
(Mean, Standard deviation)	5.61e-06(5.47e-07)	1.93e-06(2.61e-07)	4.74e-07(7.61e-08)	3.02e-06(3.37e-07)	1.42e-06(3.84e-07)
Coefficient of variation	0.0912(0.0090)	0.0282(0.0060)	0.0086(0.0028)	0.1601(0.0161)	0.3485(0.0787)
Kolmogorov–Smirnov distance	0.2697(0.0054)	0.2026(0.0038)	0.1405(0.0022)	0.3207(0.0070)	0.3863(0.0094)
Wasserstein distance	0.1308(0.0044)	0.0766(0.0028)	0.0394(0.0019)	0.0909(0.0034)	0.0540(0.0051)
Pairwise taxon-to-taxon correlation	0.0060(3.80e-04)	0.0026(2.45e-04)	9.00e-04(9.28e-05)	0.0082(3.26e-04)	0.0139(0.0022)

Schiffer, L., Azhar, R., Shepherd, L., Ramos, M., Geistlinger, L., Huttenhower, C., Dowd, J. B., Segata, N. & Waldron, L. (2019), 'Hmp16sdata: efficient access to the human microbiome project through bioconductor', *American journal of epidemiology* **188**(6), 1023–1026.

Wu, G. D., Chen, J., Hoffmann, C., Bittinger, K., Chen, Y.-Y., Keilbaugh, S. A., Bewtra, M., Knights, D., Walters, W. A., Knight, R. et al. (2011), 'Linking long-term dietary patterns with gut microbial enterotypes', *Science* **334**(6052), 105–108.

Zhou, H., He, K., Chen, J. & Zhang, X. (2022), 'Linda: linear models for differential abundance analysis of microbiome compositional data', *Genome biology* **23**(95), 1–23.
